# Interventions to support people exposed to adverse childhood experiences: systematic review of systematic reviews

**DOI:** 10.1186/s12889-020-08789-0

**Published:** 2020-05-12

**Authors:** Theo Lorenc, Sarah Lester, Katy Sutcliffe, Claire Stansfield, James Thomas

**Affiliations:** 1 Centre for Reviews & Dissemination, University of York, York, YO10 5DD UK; 2 EPPI-Centre, Social Science Research Unit, Institute of Education, University College London, 18 Woburn Square, London, WC1H 0NR UK

**Keywords:** Adverse childhood experiences, Child abuse, Resilience, psychological, Systematic review

## Abstract

**Background:**

Adverse Childhood Experiences (ACEs) such as abuse, neglect or household adversity may have a range of serious negative impacts. There is a need to understand what interventions are effective to improve outcomes for people who have experienced ACEs.

**Methods:**

Systematic review of systematic reviews. We searched 18 database sources from 2007 to 2018 for systematic reviews of effectiveness data on people who experienced ACEs aged 3–18, on any intervention and any outcome except incidence of ACEs. We included reviews with a summary quality score (AMSTAR) of 5.5 or above.

**Results:**

Twenty-five reviews were included. Most reviews focus on psychological interventions and mental health outcomes. The strongest evidence is for cognitive-behavioural therapy for people exposed to abuse. For other interventions – including psychological therapies, parent training, and broader support interventions – the findings overall are inconclusive, although there are some positive results.

**Conclusions:**

There are significant gaps in the evidence on interventions for ACEs. Most approaches focus on mitigating individual psychological harms, and do not address the social pathways which may mediate the negative impacts of ACEs. Many negative impacts of ACEs (e.g. on health behaviours, social relationships and life circumstances) have also not been widely addressed by intervention studies.

## Background

Adverse Childhood Experiences (ACEs) have been defined as stressful experiences occurring during childhood that directly impact on a child or affect the family environment in which they live. They include physical, sexual or emotional abuse, neglect, or household adversity as a result of domestic violence, imprisonment, substance abuse, parental mental health problems or family breakdown [1]. This definition was used by an influential CDC study in the 1990s; more recent work has extended the definition to include child neglect, parental bereavement, and children living in care [2]. The potential impacts of ACEs are physiological and behavioural, as well as psychological, and translate into poorer outcomes across a wide range of health domains [3, 4].

It is unclear what works to prevent or mitigate these negative consequences, or promote positive outcomes for those who have experienced ACEs. Previous reviews have looked at specific ACE populations but have not covered the whole spectrum of ACEs. The aim of this overview of systematic reviews was to provide a broad map of the evidence on the effectiveness of interventions for children and young people who have experienced ACEs, and identify gaps in the literature. It included systematic reviews including any effectiveness studies on any ACE-exposed population, and included all interventions and outcomes.

## Methods

Due to the extent of the available literature, we employed a review-of-reviews method to provide a broad overview of the topic [5, 6]. We focused on recent, high-quality reviews to prioritise the most methodologically robust evidence. The review question was: What is known from systematic reviews about the effectiveness of interventions for children and young people (3–18 years) who have been exposed to adverse childhood experiences (ACEs)?

The review protocol was registered in PROSPERO (registration CRD42018092192). EPPI-Reviewer 4 software was used to manage data. After initial ad hoc scoping searches, we searched 23 databases and other online resources that contain research literature in healthcare, mental health, social care, social science, education, child and adolescent development, and systematic reviews, as follows:

- ASSIA (Proquest)
- British Education Index (EBSCO)
- British Nursing Index (EBSCO)
- Child development and adolescent studies (EBSCO)
- CINAHL Plus (EBSCO)
- Cochrane Database of Systematic Reviews (Cochrane Library)
- Database of Reviews of Effectiveness (DARE) (Cochrane Library)
- EMBASE (OVID)
- ERIC (EBSCO)
- Health Management Information Consortium (HMIC) (OVID)
- IBSS (Proquest)
- Medline (OVID)
- PILOTS (Published International Literature On Traumatic Stress)
- PsycINFO (OVID)
- PUBMED/Medline
- Social Policy and Practice (OVID) (this includes the NSPCC Child Protection Database)
- Sociological Abstracts (Proquest)
- Social Sciences Citation Index (Web of Science)
- Bielefeld Academic Search Engine
- Campbell Collaboration Library
- Epistamonikas
- NHS Evidence
- Research in Practice

The search strategy combined terms for specific ACEs with terms for children and young people, and terms for a range of systematic review types (see Additional file 1). The search was designed and implemented by an information specialist in consultation with other members of the review team. The search was limited to reviews published since 2007; however, reviews included older primary studies. In addition, we searched for references from the NICE guideline on transition from children’s to adults’ services for young people using health or social care services (NG43) and on child abuse and neglect (NG76). Searches were carried out in March 2018.

We included systematic reviews of effectiveness data on people who experienced ACEs aged between 3 and 18 inclusive (adults who had experienced ACEs as children were also included); see Additional file 1 for details. We excluded younger children (0–2 years) as interventions focusing on this age group tend to focus on primary prevention or on psychological factors such as attachment, rather than on the impacts of ACEs. We included any outcomes measured on the person who experienced ACEs except the incidence of ACEs themselves (i.e. we did not look at the primary prevention of ACEs, or at outcomes for parents). We used the broad definition of ACEs employed by Allen and Donkin [2], with the further addition of homelessness as an important family-level stressor. We limited inclusion to reviews in English due to limited resource for translation (reviews including non-English-language primary studies were included). In more detail, the inclusion criteria were as follows:

1. Is the reference a systematic review of primary studies?
  *Include* any review of primary studies which reports some information on the search strategy and clearly defined inclusion criteria.
2. Does the review report effectiveness data?
  *Exclude* reviews of observational quantitative data or qualitative data only*.*
3. Does the review include (an) ACE population(s)?
  *Include* the following populations: sexual abuse; physical abuse; verbal or emotional abuse; neglect. *Include* household adversity, where a parent or guardian: is a victim of intimate partner violence; is in prison or on probation; has a mental health problem; abuses alcohol or drugs; is separated or divorced; or has died. *Include* children and young people living in care (in a care setting or elsewhere; *include* kinship care; *exclude* adopted children). *Include* homeless children or young people. *Exclude* reviews whose population partly overlap with our ACE criteria, and/or which is defined with broader terms such as ‘high risk’ or ‘trauma’, *unless* ≥ 70% of included studies include populations in the ACE criteria above, *or* there is a clearly presented subgroup analysis of a subset of the studies which meet these criteria.
4. Does the review concern interventions aimed at people who have experienced ACEs?
5. Does the review report outcomes for children or young people (aged 3–18 inclusive) who have experienced ACEs?
  *Include* any outcome relating to the child or young person, *except* outcomes relating to the (re)occurrence or incidence of ACEs themselves (abuse, parental substance use, homelessness etc.). *Exclude* reviews only reporting on parent/carer outcomes and not child outcomes. *Include* outcomes measured on people aged > 18 years if they experienced ACEs at age 3–18. *Include* any follow-up period.
6. Does the review report data from OECD (Organisation for Economic Co-operation and Development) member countries?
7. Is the review report available in English?
  *Include* reviews which include non-English-language primary studies, if the review itself is reported in English.
8. Was the review published in 2007 or later?
9. Is a full report of the review available?
  *Exclude* reviews for which only a protocol or an abstract is available.

An initial sample of 10% of abstracts were screened independently by two reviewers and differences resolved by discussion; the remaining abstracts were screened by a single reviewer. All full-text references were screened by two reviewers independently and differences resolved by discussion.

The AMSTAR tool was used to assess review quality [7]. Quality assessment was conducted by one reviewer and checked in detail by a second. We translated the AMSTAR results into an overall score out of 11 (see Additional file 1) and applied a threshold of 5.5 or higher for inclusion in the synthesis.

A narrative synthesis was undertaken. We grouped the data according to intervention type and outcome (see below). We extracted findings where reporting allowed, and where there was evidence from at least 2 controlled studies for a given intervention and outcome domain. Where reviews conducted meta-analyses we extracted pooled effect sizes, and otherwise summarised the results in general terms as effective, ineffective or mixed. We extracted data on all outcomes for those experiencing ACEs (not for parents), except those relating to (re)occurrence or incidence of ACEs themselves. Where multiple outcomes were reported, we aggregated them into the following domains:

- Mental health (e.g. anxiety, depression)
- Behaviour (e.g. externalising behaviour, problem behaviour)
- Social and relationship outcomes (e.g. social support).

## Results

**Fig. 1:**
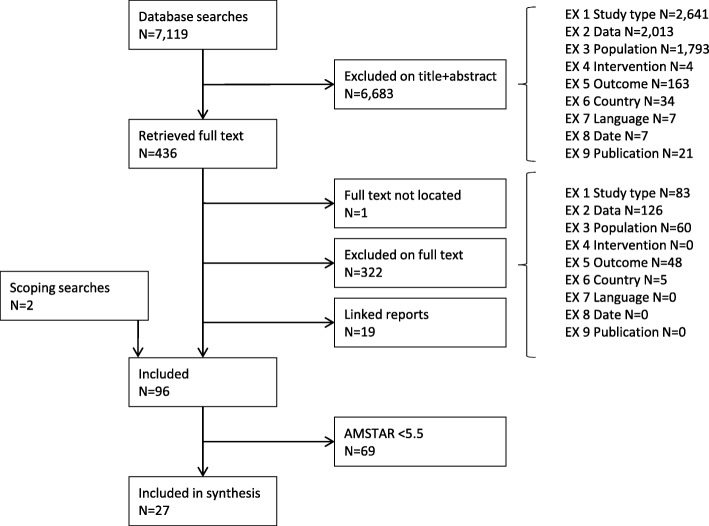
Flow of literature through the review

The flow of literature is shown in Fig. 1. Prior to quality assessment, 96 reviews were included. We included in the synthesis all reviews with an AMSTAR score of 5.5 or higher (*N* = 27). The full results of quality assessment are presented in Additional file 1. (Some reviews did not contribute to the synthesis, either because they were superseded by a later review [8] or contained no studies [9], or due to our restrictions on data extraction (see above) [10–14].)

The results are summarised in Table 1. We divided the findings into seven categories of intervention.

**Table 1:** Summary of findings (grouped by intervention type)

Reference	Ages incl	Intervention type	Population	Outcome type	Summary finding	Pooled effect size if reported	N primary studies
1. CBT							
Altena [15]	NR	Cognitive-behavioural interventions	Homeless	MH	Mixed	–	2
Beresford [16]	NR	CBT	P depression	MH	Mixed	–	2
				Behaviour	Not eff	–	3
Fraser [17]	0–17	CBT, EMDR	Abuse/neglect	MH	Mixed	–	5
				Behaviour	Mixed	–	5
Howarth [18]	< 18	CBT	P IPV	MH	Not eff	SMD − 0.43 (−1.24 to 0.50)	–
Macdonald [19]	< 18 (at ACE) < 25 (at study)	CBT	Sexual abuse	PTSD	Eff	After intervention SMD − 0.44 (−4.43 to − 1.53) At 3–6 mo SMD − 0.39 (− 0.74 to − 0.04)) At ≥1 y SMD − 0.38 (− 0.65 to − 0.11)	6
				Anxiety	Eff	After intervention SMD − 0.23 (− 0.42 to − 0.03) At 3–67 mo SMD − 0.38 (95% CI − 0.61 to − 0.14) At ≥1 y SMD − 0.28 (− 0.52 to − 0.04)	5
				Sexualised behaviour	Not eff	After intervention MD − 0.65 (−3.53 to 2.24) At 3–6 mo MD − 0.46 (−5.68 to 4.76) At ≥1 y MD − 1.61 (− 5.72 to 2.49)	5
				Externalising behaviour	Not eff	After intervention SMD − 0.12 (− 0.40 to 0.17) At 3–6 mo SMD − 0.11 (− 0.42 to 0.21) At ≥1y SMD 0.05 (− 0.16 to 0.27)	7
				Depression	Mixed	After intervention MD −2.83 (−4.53 to −1.13) At 3–6 mo MD − 1.76 (−3.33 to −0.20) At ≥1 y MD − 1.42 (− 2.91 to 0.06)	5
			Other abuse/neglect	MH	Eff	–	5
				Behaviour	Mixed	–	5
Wethington [20]	≤21	CBT	Sexual abuse	Anxiety	Not eff	SMD −0.23 (−0.48 to 0.01)	4
				Depression	Not eff	SMD −0.03 (−0.28 to 0.21)	4
				PTSD	Not eff	SMD −0.29 (−0.69 to 0.11)	4
Wilen [21]	NR (at ACE) > 18 (at study)	CBT	Sexual abuse	PTSD	Not eff	SMD − 0.06 (− 0.49 to 0.37)	2
2. Other psychological therapies							
Altena [15]	NR	Brief motivational interviewing	Homeless	Drug/alcohol use	Not eff	–	2
BC Centre [22]	NR	Mother-child psychotherapy	P IPV	Behaviour	Eff	–	2
				MH	Eff	–	2
Howarth [18]	< 18	Psychotherapy	P IPV	MH	Not eff	SMD −0.51 (−1.13 to 0.10)	–
Kinsey [23]	NR	Relational psychological interventions	LACYP	Behaviour	Mixed	–	2
Macdonald [19]	< 18 (at ACE) < 25 (at study)	Group activity-based therapies	Abuse/neglect	MH	Mixed	–	3
		Multisystemic family therapy		MH	Eff	–	3
		Family-based systemic interventions		Behaviour	Mixed	–	3
		Group therapy		MH	Eff	–	2
				Behaviour	Mixed	–	3
		Psychotherapy and counselling		MH	Mixed	–	1
				Behaviour	Mixed	–	4
Wilen [21]	NR (at ACE) > 18 (at study)	Humanistic therapy	Sexual abuse	PTSD	Not eff	SMD −0.14 (− 0.53 to 0.26)	3
3. Psychoeducation							
Howarth [18]	< 18	Psychoeducation (child + parent)	P IPV	MH	Not eff	SMD 0.05 (− 0.43 to 0.50)	–
				Behaviour	Not eff	SMD −0.18 (− 0.57 to 0.23)	–
		Psychoeducation (child only)		MH	Not eff	SMD −0.39 (− 0.80 to 0.02)	–
				Behaviour	Not eff	SMD 0.27 (−0.21 to 0.77)	–
Loechner [24]	≤18	Group psychoeducation	P depression	Depression (incidence)	Eff	At 6–15 mo RR 0.56 (0.40 to 0.77)	4
				Depressive symptoms	Mixed	At 0–4 mo SMD −0.20 (− 0.34 to − 0.06)) At 5–12 mo SMD − 0.11 (− 0.25 to 0.03) At 15–72 mo SMD − 0.05 (− 0.18 to 0.08)	6
Macdonald [19]	< 18 (at ACE) < 25 (at study)	Group psychoeducation	Abuse/neglect	MH	Mixed	–	7
				Behaviour	Not eff	–	2
4. Parent / foster carer training							
BC Centre [22]	NR	Parent training + child therapy	P IPV	Behaviour	Eff	–	4
				MH	Mixed	–	2
Everson-Hock b [25]	Any	Foster carer training	LACYP	Behaviour	Mixed	–	3
Fraser [17]	0–14	Foster carer training	LACYP	Behaviour	Mixed	–	4
Howarth [18]	< 18	Parent training + advocacy	P IPV	Behaviour	Eff	SMD −0.46 (− 0.85 to −0.06)	–
				MH	Not eff	SMD −0.31 (−1.04 to 0.46)	–
Kemmis-Riggs [26]	NR	Foster carer training	LACYP	Behaviour	Mixed	–	12
				Social	Mixed	–	3
Kinsey [23]	NR	Foster carer training	LACYP	Behaviour	Mixed	–	5
Macdonald [19]	< 18 (at ACE) < 25 (at study)	Parent-child interaction therapy	Abuse/neglect	Behaviour	Mixed	–	2
Montgomery [27]	0–18	Parent-child interaction therapy	Physical abuse	Behaviour	Mixed	–	2
Troy [28]	3–18	Parent training	P in criminal justice system	Behaviour	Not eff	–	4
				MH	Mixed	–	2
Turner [29]	≤18	Group cognitive-behavioural foster carer training	LACYP	MH	Not eff	SMD 0.13 (− 0.71 to 0.96)	2
				Behaviour	Not eff	SMD 0.23 (−0.06 to 0.52)	2
Ziviani [30]	0–18	Foster carer training	LACYP	Behaviour	Not eff	–	2
5. Cross-sector support							
Howarth [18]	< 18	Advocacy	P IPV	MH	Not eff	SMD 0.07 (−0.23 to 0.38)	–
				Behaviour	Not eff	SMD 0.18 (− 0.11 to 0.47)	–
Jones [31]	NR	Case management	LACYP	Service receipt	Not eff	–	3
Kinsey [23]	NR	‘Wraparound’ support	LACYP	MH	Eff	–	3
				Behaviour	Mixed	–	2
Macdonald [19]	< 18 (at ACE) < 25 (at study)	Treatment foster care	Abuse/neglect	Behaviour	Mixed	–	4
				MH	Mixed	–	2
				Social	Not eff	–	2
Ziviani [30]	0–18	Case management	LACYP	Behaviour	Eff	–	2
				Social	Eff	–	2
				Criminal behaviour	Mixed	–	2
6. Educational interventions							
Evans [32]	≤18	Educational interventions	LACYP	Academic skills	Mixed		9
				School attendance	Mixed		3
Macdonald [19]	< 18 (at ACE) < 25 (at study)	Therapeutic day care	Abuse/neglect	MH	Eff		2
Montgomery [27]	0–18	Therapeutic day care	Physical abuse	Social	Eff		2
7. Housing / life skills							
Coren [33]	0–24	Various interventions	Homeless	Alcohol or drug use	Not eff	Days alcohol use at 3 mo MD −0.56 (−1.13 to 0.01) Days alcohol use at 6 mo MD 1.05 (−1.76 to 3.86) Days alcohol use at 12 mo MD 0.63 (−2.23 to 3.48) Drinking index score at 3 mo MD 1.08 (−4.42 to 6.57) % days drug/alcohol use at 3 mo MD − 0.70 (−9.09 to 7.70) % days drug/alcohol use at 6 mo MD − 2.15 (−9.82 to 5.53) % days drug/alcohol use at 12 mo MD 5.87 (=5.06 to 16.79) % days drug use at 3 mo MD 0.67 (−6.82 to 8.15) % days drug use at 6 mo MD − 2.28 (− 11.53 to 6.96) % days drug use at 12 mo MD −5.28 (− 13.79 to 3.23)	11
				Sexual behaviour	Not eff	N times had sex at 3 mo MD −0.51 (−1.13 to 0.10) N times had sex at 6 mo MD − 0.04 (− 0.22 to 0.13) N partners at 3 mo MD − 0.56 (− 1.13 to 0.01) N partners at 6 mo − 0.56 (− 1.13 to 0.01)	5
				MH	Not eff	Self−esteem SMD 0.11 (−0.22 to 0.44) Depression at 3 mo SMD − 0.03 (− 0.22 to 0.17) Depression at 6 mo SMD 0.83 (− 0.88 to 2.55) Depression at 12 mo SMD 1.28 (− 0.36 to 2.92)	7
				Delinquency	Mixed	At 3 mo SMD −0.29 (− 0.54 to − 0.03) At 6 mo SMD − 0.07 (− 0.52 to 0.37) At 12 mo 0.31 (−1.58 to 2.20)	2
Everson-Hock a [34]	NR	Support for transition from care	LACYP	Housing / independent living	Eff	–	6
				Educational	Not eff	–	5
				Employment	Not eff	–	6
				Crime	Not eff	–	6
				Homelessness	Not eff	–	4
				MH	Not eff	–	3

*Abbreviations*: *CBT* Cognitive behavioural therapy, *Eff* Effective, *EMDR* Eye movement desensitation and reprocessing, *IPV* Intimate partner violence, *LACYP* Looked-after children and young people, *MD* Mean difference, *MH* Mental health, *P* parent(al), *PTSD* Post-traumatic stress disorder, *RR* Risk ratio, *SMD* Standardised mean difference

1. Cognitive Behavioural Therapy. Seven reviews investigated CBT for a range of ACE populations [15–21]. The most substantive results come from Macdonald et al.’s review, which found that CBT improved mental health outcomes for people who had experienced abuse or neglect [19].

2. Other psychological therapies. A range of other psychological therapies, such as brief motivational interviewing, family therapy and psychodynamic psychotherapy, were evaluated in six reviews [15, 18, 19, 21–23]. The findings do not provide strong evidence of effectiveness, although the interventions are heterogeneous.

3. Psychoeducation. Three reviews included psychoeducation [18, 19, 24]; the findings are mixed, although one meta-analysis finds evidence of effectiveness for mental health outcomes in children of parents with depression [24].

4. Parent / foster carer training. Eleven reviews included training for parents and foster carers, including a range of ACE populations; most studies focus on behaviour problems [17–19, 22, 23, 25–30]. However, the results overall are inconclusive.

5. Cross-sector support. Five reviews included cross-sector support interventions (such as case management, ‘wraparound’ support and treatment foster care), mainly for looked-after children and young people [18, 19, 23, 30, 31]. This category is heterogeneous and the results overall are mixed, but there are some positive findings.

6. Educational interventions. The evidence on educational and school-based interventions mainly comes from a single review on looked-after children and young people; the results overall are mixed [32].

7. Housing and life skills interventions. One review finds that support services for young people transitioning out of care are effective for housing and independent living outcomes but not for other outcomes [34], and one that interventions for homeless young people are largely ineffective for outcomes including alcohol or drug use or mental health [33].

## Discussion

The findings of this review of reviews indicate that there is limited evidence for the effectiveness of most interventions for children and young people who have experienced ACEs. The strongest evidence is for the effectiveness of CBT for mental health outcomes in children who have been sexually abused. The evidence on other interventions and populations is generally more equivocal, although there are some positive findings.

Our findings indicate some important gaps in the review-level evidence. Most data relate to psychological interventions aiming to improve individuals’ mental resilience; this is true across the abuse and neglect populations as well as the household adversity populations. For the looked-after and homeless populations the range of interventions is somewhat broader, and includes more service-level programmes aiming to improve the support provided by agencies such as welfare services or schools. While the evidence on these interventions is inconclusive, it may be of value to explore their generalisability to other ACE populations. There is very limited evidence concerning any social or community-level interventions, for example to address socio-economic disadvantage or social isolation; this is not unexpected, as most such interventions include a broad range of populations and are unlikely to be evaluated on ACE populations specifically, but it is a limitation.

Similarly, the great majority of the outcomes measured in the studies relate to mental health or, for younger children, behaviour problems. With the exception of looked-after and homeless children and young people, there is very little data on broader outcomes in the domains of social relationships, life circumstances (e.g. housing, education) or behaviours (e.g. drug use, criminal involvement). As these outcomes have been identified in epidemiological research as important negative impacts of ACEs, the lack of effectiveness data on them is an important gap in the evidence.

The methodology used for this review has some limitations. It involves some double-counting of primary studies. In most cases the actual overlap between reviews is fairly limited and, given the high-level nature of the synthesis, unlikely to have a major impact on the interpretation of findings. The main exception is the literature on foster parent training, where three different reviews show substantial overlap [17, 23, 26]. The findings on this intervention should therefore be treated with caution. Our findings represent only a very high-level overview of the data: some of the intervention categories are very broad, and the ‘mixed’ results call for more detailed exploration. The findings are also partly dependent on review authors’ categorisation of interventions.

We defined ACEs in terms of a fixed list of population characteristics, partly for pragmatic reasons and partly because this approach has been widely used, and so facilitates comparison of our findings with the broader literature. However, it has some conceptual limitations. Many potentially relevant life stressors are not captured in ACE on our definition, including for example: factors affecting children and young people directly such as mental or physical health conditions, or alcohol or drug abuse; socioeconomic disadvantage and poverty, both at family level and community level; or broader environmental stressors such as community violence or natural disaster. The broader concept of ‘trauma’ covers some of these stressors [20, 35, 36], and could usefully be used to illuminate the broader dynamics of stress and resilience at work in ACE populations, although it arguably has limitations of its own. Conversely, treating ACEs as an itemised list of discrete experiences may not capture the role of multiple cumulative stress. Experiencing multiple ACEs is much more strongly correlated with negative outcomes than experiencing one or two [3, 37]. However, as our findings show, the effectiveness literature has tended to treat ACE populations separately, with limited consideration of the impacts of multiple interacting forms of disadvantage.

These limitations aside, this review suggests that the evidence base on interventions for people who have experienced ACEs may not be ideally suited to informing policy and practice. While individual interventions to mitigate psychological trauma are a potentially important avenue for addressing ACEs, many would argue that they need to form part of broader strategies which also aim to address the social factors which may mediate the negative impacts of ACEs, including material disadvantage and social isolation [2]. Such strategies could draw on interventions for the primary prevention of ACEs which were not included in this review [38, 39], but evidence on what works for people who have experienced ACEs is also needed. This could take the form of focused evaluation studies on, for example, school-based programmes or life skills training aiming to empower ACE populations, and/or subgroup analyses to understand how community-level interventions might address the consequences of ACEs. More indirect approaches could also be of value, such as modelling work using the epidemiological evidence base on the prevalence and distribution of ACEs to understand the likely impact of interventions with a broader population focus. Of course, such work should be based on consultation and involvement of children and young people who have experienced ACEs, to ensure it addresses their needs. It could also draw on the extensive body of qualitative evidence on these questions which points to the need for (evaluations of) longer term interventions which afford the necessary time to build up trust and address the ongoing and multi-faceted needs of children and young people affected by ACEs [40].

## Conclusions

The evidence for most interventions for people who have experienced Adverse Childhood Experiences is equivocal; the most promising results are for CBT for mental health outcomes. The majority of the existing evidence focuses on psychological interventions and on mental health outcomes, and there is a lack of studies on service- or community-level interventions, and on social or behavioural outcomes. Evidence from observational and qualitative research indicates that people exposed to ACEs, especially multiple ACEs, often have complex needs, but there is limited information in the evaluation literature about how best to address these needs.

## Supplementary information

**Additional file 1.Appendix A**. Example search strategy. **Appendix B**. Quality assessment tool. **Appendix C**. Results of quality assessment. **Appendix D**. Quality assessment tools used in included reviews.

## Data Availability

The datasets used and/or analysed during the current study are available from the corresponding author on reasonable request.

## Abbreviations

ACEs: Adverse Childhood Experiences
AMSTAR: A MeaSurement Tool to Assess systematic Reviews
CBT: Cognitive behavioural therapy
CDC: Centers for Disease Control and Prevention
NICE: National Institute for Health and Care Excellence
