# Supplementary material for: Interventions to support people exposed to adverse childhood experiences: systematic review of systematic reviews
Source: BMC Public Health. 2020 May 12;20:657. doi: 10.1186/s12889-020-08789-0 (PMC7216383; doi:10.1186/s12889-020-08789-0)
Supplement: Supplementary file 1 — Additional file 1.Appendix A. Example search strategy. Appendix B. Quality assessment tool. Appendix C. Results of quality assessment. Appendix D. Quality assessment tools used in included reviews. [file 12889_2020_8789_MOESM1_ESM.docx]

Interventions to support people exposed to Adverse Childhood Experiences: systematic review of systematic reviews

Methodological appendices

**Appendix A. Example search strategy**

The search strategy below was used in the MEDLINE database. A translated version was used in the other databases. [Note that the search was initially designed to capture all reviews, not just reviews of effectiveness.]

Databases: Ovid MEDLINE(R) Epub Ahead of Print, In-Process & Other Non-Indexed Citations, Ovid MEDLINE(R) Daily and Ovid MEDLINE(R) <1946 to Present>

Date searched: 22/3/18

No. of records: 1,990

| **Set** | **Searches** |
| --- | --- |
| **1** | ((divorce* and (parent* or child* or family or families)) or (parent* adj2 separat*) or (marital adj2 separation*) or (family adj2 breakdown) or (family adj2 breakup) or (family adj2 separation) or (marital adj2 break*) or (marriage adj2 break*)).ti,ab |
| **2** | Divorce/ |
| **3** | 1 or 2 |
| **4** | ("parentally bereaved" or "parental bereavement" or "Parental death" or "bereaved children" or "parental loss" or "loss of a parent" or "childhood bereavement" or (children* adj grief) or (grieving adj child*) or ((parent* or mother* or father* or carer* or caregiver*) adj3 death)).ti,ab |
| **5** | (parental death/ or maternal death/) not (infant death/ or pregnancy/ or "cause of death"/ or perinatal death/ or exp "abortion, induced"/) |
| **6** | 4 or 5 |
| **7** | exp mental disorders/ and "Parent-Child relations"/ |
| **8** | ((parent* or mother* or father* or carer? or caregiver?) adj3 "mental health" adj (problem* or condition* or disorder* or illness* or difficult*)).ti,ab. |
| **9** | ((parent* or mother* or father* or carer? or caregiver?) adj3 (mental* adj ill*)).ti,ab |
| **10** | ((parent* or mother* or father* or carer? or caregiver?) adj3 (depressi* or anxiety) adj3 (clinical or severe or major or chronic* or illness* or condition* or disorder* or difficult*)).ti,ab. |
| **11** | ((parent* or mother* or father* or carer? or caregiver?) adj3 depression).ti,ab. |
| **12** | ((parent* or mother* or father* or carer? or caregiver?) adj3 (suicidal or suicide)).ti,ab. |
| **13** | ((parent* or mother* or father* or carer or caregiver) adj3 (mental* adj disorder*)).ti,ab. |
| **14** | ((parent* or mother* or father* or carer or caregiver) adj3 (psychiatric or psychologic*) adj3 (illness* or condition* or disorder* or difficult*)).ti,ab |
| **15** | 7 or 8 or 9 or 10 or 11 or 12 or 13 or 14 |
| **16** | ((parent* or mother* or father* or carer* or caregiver* or sibling* or "family member" or brother* or sister*) adj3 (incarcerat* or prison* or "imprisoned" or "imprisonment" or jail* or "penitatiary" or criminal* or "detention" or "probatation" or "parole" or "young offender" or "young offenders")).ti,ab. |
| **17** | ("children of" adj2 prisoners).ti,ab. |
| **18** | ((parent* or mother* or father* or carer* or caregiver* or sibling* or "family member" or brother* or sister*) adj2 (criminal* adj1 convict*)).ti,ab. |
| **19** | (Parent-child relations/ or child welfare/) and (Prisoners/ or Prisons/) |
| **20** | 16 or 17 or 18 or 19 |
| **21** | ((parent* or mother* or father* or carer? or caregiver?) adj3 (substance? or drug? or drinking or alcohol* or solvent? or medication?) adj2 ("use" or abus* or misuse* or addict* or disorder* or dependen*)).ti,ab. |
| **22** | ((parent* or mother* or father* or carer? or caregiver?) adj3 (alcoholism or alcoholic* or "heavy drinking" or addicts or "heavy drinkers" or "substance related" or "substance affected" or (drinking adj1 problem*))).ti,ab. |
| **23** | exp Substance-Related Disorders/ and Parent-Child relations/ |
| **24** | 21 or 22 or 23 |
| **25** | ("Domestic violence" or "intimate partner violence" or ("IPV" not (vaccin* or ventilat*)) or "intimate partner abuse" or (battered adj3 (mother* or father* or spouse or partner)) or "domestic abuse" or "partner abuse" or (family adj2 violen*) or (families adj2 violen*)).ti,ab. |
| **26** | domestic violence/ or spouse abuse/ or intimate partner violence/ |
| **27** | 25 or 26 |
| **28** | (((Sexual or physical* or verbal* or emotional* or psychologic*) adj2 (Abus* or assault*)) or incest* or "sexual violence" or ((rape or raped or violence) adj4 (home or family or parent* or families or homes or household?))).ti,ab. |
| **29** | Exposure to Violence/ or child abuse, sexual/ or physical abuse/ |
| **30** | (abus* or assault*).ti,ab. |
| **31** | (((Ill adj treat*) or "Ill treatment" or maltreat* or mistreat* or "Neglect" or "cruelty" or "cruel" or abus* or assault*) adj3 ("offspring" or "young people" or children* or "childhood" or "child" or "boys" or "girls" or adolescen* or youth* or "young person" or teen* or preadolescen* or "early life")).ti,ab. |
| **32** | maternal deprivation/ or Paternal deprivation/ |
| **33** | (victim* adj2 ("home" or "homes" or "family" or "families" or "household" or "households")).ti,ab. |
| **34** | Child Abuse/ |
| **35** | "Child of Impaired Parents"/ or "Child, Foster"/ or "Child, Orphaned"/ or "Child, Adopted"/ or Homeless Youth/ |
| **36** | (homeless* adj3 ("young people" or children* or childhood or child or boys or girls or adolescen* or youth* or "young person*" or teen*)).ti,ab. |
| **37** | (("Looked after" or foster* or "adoptive" or "in care") adj1 ("young people" or children* or childhood or child or boys or girls or adolescen* or youth* or "young person*" or teen*)).ti,ab. |
| **38** | ((("moving" adj1 "care") or ("leaving" adj1 "care")) and ("young people" or children* or childhood or child or boys or girls or adolescen* or youth* or "young person" or teen*)).ti,ab. |
| **39** | "Foster Home Care"/ |
| **40** | ("care leaver?" or "residential child*" or ("child welfare" adj2 (service* or centre or centres or center or centers)) or ("child protection" adj2 (service* or center or centers or centre or centres))).ti,ab. |
| **41** | (("living in care" or "kinship care" or "foster care" or "adoption care" or "group home" or "group homes" or "out of home placement" or "out of home care" or "child placement" or "local authority care" or "state care" or "alternative care" or "kith and kin care" or "kinship care") and ("young people" or children* or childhood or child or boys or girls or adolescen* or youth* or "young person" or teen*)).ti,ab. |
| **42** | adult survivors of child abuse/ or Adult Survivors of Child Adverse Events/ |
| **43** | child/ or "child, preschool"/ or adolescent/ |
| **44** | (offspring or "young people" or children* or childhood or child or boys or girls or adolescen* or youth* or "young person" or teen* or juvenile* or preadolescen* or "early life").ti,ab |
| **45** | 43 or 44 |
| **46** | "Caregivers"/ or parents/ or parenting/ or fathers/ or mothers/ or exp "Parent-Child Relations"/ or exp "Nuclear Family"/ or "Family"/ or exp "Family Relations"/ or exp "Grandparents"/ or exp "Single-Parent Family"/ |
| **47** | ("Parent" or "parents" or "mother" or "mothers" or "father" or "fathers" or "home" or "homes" or "household" or "households" or "family" or "primary carer" or "foster carer" or "guardian" or "guardians" or "grandparent" or "grandparents" or "relatives" or maternal* or paternal* or sibling* or grandfather* or grandmother* or caregiver* or carer? or "families").ti,ab. |
| **48** | 46 or 47 |
| **49** | ((child* or "young people*" or adolesc*) and (abus* or neglect)).jw. |
| **50** | (abus* or neglect).jw. |
| **51** | ("Stressful childhood experiences" or "adverse childhood events" or "adverse childhood experiences" or "traumatic childhood experiences" or "Stressful childhood experience" or "adverse childhood event" or "adverse childhood experience" or "traumatic childhood experience" or "adverse home environment" or "adverse home environments" or "adverse family environment" or "adverse family environments" or "stressful home environment" or "stressful home environments" or "stressful family environment" or "stressful family environments").ti,ab. |
| **52** | ((("synthesis" or "systematic") and ("evidence" or "research" or "review")) or ("review" and (integrat* or critical* or "mapping" or "comprehensive" or "evidence" or "research" or "literature"))).ti. or ((systematic adj2 review*) or ("meta-analysis" or "Review articles" or "systematic review*" or "Overview of reviews" or "Review of Reviews") or ("data synthesis" or "evidence synthesis" or "metasynthesis" or "meta-synthesis" or "narrative synthesis" or "qualitative synthesis" or "quantitative synthesis" or "realist synthesis" or "research synthesis" or "synthesis of evidence" or "thematic synthesis" or "systematic map*" or "metaanaly*" or "meta-analy*" or "systematic overview*" or "systematic review*" or "systematically review*" or "bibliographic search" or "database search" or "electronic search" or "handsearch*" or "hand search*" or "keyword search" or "literature search" or "search term*" or "literature review" or "overview of reviews" or "review literature" or "reviewed the literature" or "reviews studies" or "scoping stud*" or "overview study" or "meta-ethnograph*" or "meta-epidemiological" or "data extraction" or "meta-regression" or "narrative review" or "art review" or "scoping review" or "iterative review" or "meta-summary")).ti,ab. |
| **53** | 30 and 45 and 48 |
| **54** | 3 or 6 or 15 or 20 or 24 or 27 or 28 or 29 or 32 or 33 or 50 |
| **55** | 45 and 54 |
| **56** | 31 or 34 or 35 or 36 or 37 or 38 or 39 or 40 or 41 or 42 or 49 or 51 or 53 |
| **57** | 55 or 56 |
| **58** | limit 57 to systematic reviews |
| **59** | 52 and 57 |
| **60** | 58 or 59 |
| **61** | 60 not (animals/ not (animals/ and humans/)) |
| **62** | limit 61 to yr="2007 -Current" |
| **63** | limit 62 to english |

**Appendix B. Quality assessment tool**

Our quality assessment procedure was based on the AMSTAR tool (Shea et al., 2017). This tool contains 16 items. For the purposes of this review, the questions in the full tool relating to meta-analysis (questions 11, 12, 14, and 15) were not used in formulating a quality score, as not all reviews reported meta-analysis and so these questions were often not applicable. We also did not include question 3 (“Did the review authors explain their selection of the study designs for inclusion in the review?”) as often this was implicitly stated rather than explicitly explained within the reviews. This left N=11 questions, giving each review a score between 0 and 11 (scored as Yes=1, Partial Yes=0.5, No=0).

**Appendix C. Results of quality assessment**

Table 1. Results of AMSTAR quality assessment (N=96)

| **Reference** | **1** | **2** | **3** | **4** | **5** | **6** | **7** | **8** | **9** | **10** | **11** | **12** | **13** | **14** | **15** | **16** | **Score /11** |
| --- | --- | --- | --- | --- | --- | --- | --- | --- | --- | --- | --- | --- | --- | --- | --- | --- | --- |
| Al et al. (2012) | Y | N | Y | P | Y | Y | N | P | Y | N | Y | N | N | Y | Y | N | 5 |
| Altena et al. (2010) | Y | N | N | P | Y | N | N | Y | Y | N | X | X | Y | X | X | Y | 6.5 |
| Bassuk et al. (2014) | Y | Y | N | P | Y | Y | N | Y | Y | N | X | X | Y | X | X | N | 7.5 |
| Bee et al. (2014) | Y | Y | Y | Y | Y | Y | Y | Y | Y | N | Y | Y | Y | Y | Y | Y | 10 |
| Beresford et al. (2008) | Y | P | N | Y | Y | Y | N | Y | Y | N | X | X | Y | X | X | Y | 8.5 |
| Bergman et al. (2017) | Y | N | N | P | Y | N | N | Y | P | N | X | X | N | X | X | Y | 5 |
| British Columbia Centre (2013) | Y | N | N | Y | Y | Y | Y | Y | Y | Y | X | X | Y | X | X | N | 9 |
| Broning et al. (2012) | Y | N | N | P | Y | N | N | Y | Y | N | X | X | Y | X | X | Y | 6.5 |
| Byrne (2017) | Y | N | N | P | Y | N | N | N | N | N | X | X | N | X | X | Y | 3.5 |
| Calhoun (2015) | Y | N | N | P | N | N | N | P | N | N | X | X | N | X | X | N | 2 |
| Chen and Panebianco (2018) | Y | N | N | P | Y | N | N | P | N | N | X | X | N | X | X | N | 3 |
| Chronis-Tuscano et al. (2017) | Y | N | N | P | N | N | N | P | N | N | X | X | N | X | X | N | 2 |
| Corcoran and Pillai (2008) | Y | N | N | Y | N | Y | N | P | Y | N | Y | N | N | Y | N | N | 4.5 |
| Coren et al. (2016) | Y | Y | N | Y | Y | Y | Y | Y | Y | Y | Y | N | Y | Y | N | Y | 11 |
| Cunha (2008) | Y | N | N | P | N | Y | N | N | N | N | Y | N | N | N | N | N | 2.5 |
| Currier et al. (2007) | Y | N | Y | P | N | N | N | N | N | N | Y | N | N | Y | N | N | 1.5 |
| Davies and Allen (2017) | Y | N | N | P | N | N | N | N | P | N | X | X | N | X | X | N | 2 |
| Dawson and Jackson (2013) | Y | N | N | P | N | N | N | N | Y | N | X | X | N | X | X | N | 2.5 |
| Dorrepaal et al. (2014) | Y | N | N | N | N | N | N | P | N | N | Y | N | N | N | N | Y | 2.5 |
| Downes et al. (2016) | Y | N | N | P | Y | N | N | N | N | N | X | X | N | X | X | N | 2.5 |
| Ehring et al. (2014) | Y | N | N | P | N | Y | N | P | Y | N | Y | N | N | Y | Y | Y | 5 |
| Evans et al. (2017) | Y | N | N | Y | Y | Y | N | P | Y | N | X | X | N | X | X | N | 5.5 |
| Everson-Hock et al. (2011) | Y | N | N | Y | Y | Y | N | P | Y | N | X | X | Y | X | X | N | 6.5 |
| Everson-Hock et al. (2012) | Y | N | N | P | Y | Y | N | P | Y | N | X | X | Y | X |  | N | 6 |
| Forsman and Vinnerljung (2012) | Y | N | Y | Y | N | N | N | P | N | N | X | X | N | X | X | N | 2.5 |
| Fraser et al. (2013) | Y | Y | N | Y | Y | Y | Y | P | Y | Y | X | X | Y | X | X | Y | 10.5 |
| Furr-Roeske (2011) | Y | N | N | P | N | N | N | P | P | N | X | X | N | X | X | N | 2.5 |
| Gunlicks and Weissman (2008) | Y | N | N | P | N | N | N | P | N | N | X | X | N | X | X | Y | 3 |
| Hackett (2013) | Y | N | N | P | N | N | N | N | N | N | Y | N | N | N | N | N | 1.5 |
| Hambrick et al. (2016) | Y | P | N | P | Y | Y | N | N | Y | N | X | X | N | X | X | N | 5 |
| Harvey and Taylor (2010) | Y | N | Y | P | Y | Y | Y | N | N | N | Y | N | N | Y | Y | N | 4.5 |
| Herbert and Bromfield (2016) | N | N | N | P | N | N | N | P | N | N | X | X | N | X | X | Y | 2 |
| Hetzel-Riggin et al. (2007) | Y | N | N | P | N | Y | N | N | P | N | Y | N | N | Y | Y | N | 3 |
| Holtzhausen et al. (2016) | Y | N | N | P | N | N | N | N | N | N | X | X | N | X | X | N | 1.5 |
| Hooker et al. (2016) | Y | N | N | Y | N | N | N | N | N | N | X | X | N | X | X | N | 2 |
| Howarth et al. (2016) | Y | Y | N | P | Y | Y | Y | Y | Y | N | Y | N | Y | Y | N | Y | 9.5 |
| Jensen de López et al. (2017) | Y | N | N | P | Y | N | N | P | N | N | X | X | N | X | X | Y | 4 |
| Jones et al. (2008) | Y | N | N | Y | Y | Y | P | Y | Y | Y | X | X | Y | X | X | N | 8.5 |
| Journot-Reverbel et al. (2017) | Y | N | N | P | N | N | N | P | N | N | X | X | N | X | X | Y | 3 |
| Kanine et al. (2015) | Y | N | N | P | N | Y | N | P | Y | N | X | X | Y | X | X | N | 5 |
| Kemmis-Riggs et al. (2018) | Y | Y | N | P | Y | Y | N | P | Y | N | X | X | N | X | X | N | 6 |
| Kerr and Cossar (2014) | Y | N | N | Y | N | Y | N | P | Y | N | X | X | N | X | X | N | 4.5 |
| Kim et al. (2016) | Y | N | N | P | Y | Y | N | P | Y | N | X | X | N | X | X | N | 5 |
| Kinsey and Schlosser (2013) | Y | N | N | P | N | Y | Y | P | Y | N | X | X | N | X | X | Y | 6 |
| Korotana et al. (2016) | Y | N | N | P | N | N | N | P | N | N | X | X | N | X | X | N | 2 |
| Kowalik et al. (2011) | Y | N | N | P | N | N | N | P | N | N | Y | N | N | Y | Y | N | 2 |
| Leenarts et al. (2013) | Y | N | N | P | Y | Y | N | P | Y | N | X | X | N | X | X | Y | 6 |
| Leve et al. (2012) | Y | N | N | N | N | N | N | P | N | N | X | X | N | X | X | Y | 2.5 |
| Liabo et al. (2013) | Y | N | N | Y | Y | N | N | P | N | N | X | X | N | X | X | N | 3.5 |
| Loechner et al. (2017) | Y | N | N | Y | N | Y | N | P | Y | N | Y | N | N | Y | Y | Y | 5.5 |
| Macdonald et al. (2012) | Y | Y | N | Y | Y | Y | Y | Y | Y | N | Y | N | N | Y | N | Y | 9 |
| Macdonald et al. (2016) | Y | Y | N | Y | Y | Y | Y | Y | Y | N | Y | N | Y | Y | N | Y | 10 |
| Mannay et al. (2015) | Y | N | N | Y | Y | N | N | P | Y | N | X | X | N | X | X | N | 4.5 |
| Marsh (2017) | Y | N | N | P | N | N | N | N | N | N | Y | N | N | Y | Y | N | 1.5 |
| McDonnell and Garbers (2017) | Y | N | N | N | Y | N | N | N | N | N | X | X | N | X | X | N | 2 |
| McMillan et al. (2008) | Y | N | N | Y | N | Y | P | P | Y | N | X | X | N | X | X | N | 5 |
| Miffitt (2014) | Y | N | N | P | N | N | N | N | N | N | X | X | N | X | X | N | 1.5 |
| Montgomery et al. (2009) | Y | N | Y | Y | N | N | N | P | N | Y | X | X | Y | X | X | N | 5.5 |
| Naranbhai et al. (2011) | Y | Y | N | Y | Y | Y | Y | Y | Y | N | X | X | Y | X | X | Y | 10 |
| Niccols et al. (2012) | Y | N | N | Y | N | Y | N | N | Y | N | X | X | N | X | X | N | 4 |
| O'Haire et al. (2015) | Y | P | N | P | N | N | N | P | N | N | X | X | N | X | X | N | 2.5 |
| Parker and Turner (2013) | Y | Y | Y | Y | Y | N | Y | N | Y | N | X | X | N | X | X | Y | 7 |
| Passarela et al. (2010) | Y | N | N | P | N | N | N | P | N | N | X | X | N | X | X | N | 2 |
| Phillips et al. (2009) | Y | N | N | P | N | N | N | N | N | N | X | X | N | X | X | N | 1.5 |
| Poli et al. (2017) | Y | N | N | P | N | Y | N | P | N | N | X | X | N | X | X | N | 3 |
| Premji et al. (2007) | Y | N | N | Y | N | N | N | P | Y | N | X | X | N | X | X | N | 3.5 |
| Reupert et al. (2013) | Y | N | N | P | N | N | N | P | N | N | X | X | N | X | X | Y | 3 |
| Rizo et al. (2011) | Y | N | N | P | N | N | N | P | N | N | X | X | N | X | X | N | 2 |
| Roberts et al. (2016) | Y | N | N | Y | N | N | N | P | Y | N | X | X | N | X | X | N | 3.5 |
| Rosner et al. (2010) | Y | N | N | Y | Y | N | N | P | N | N | Y | N | N | Y | Y | N | 3.5 |
| Rubin et al. (2017) | Y | N | Y | P | N | N | N | N | N | N | Y | N | N | N | N | Y | 2.5 |
| Ruff et al. (2010) | Y | N | N | P | N | N | N | N | N | N | X | X | Y | X | X | N | 2.5 |
| Sanchez-Meca et al. (2011) | Y | N | N | Y | N | Y | N | N | Y | N | N | N | N | Y | Y | N | 4 |
| Siegenthaler et al. (2012) | Y | N | N | Y | N | N | N | N | Y | N | Y | N | N | Y | Y | N | 3 |
| Silverman et al. (2008) | Y | N | N | P | Y | N | N | N | Y | N | Y | N | N | N | N | N | 3.5 |
| Slesnick et al. (2009) | Y | N | N | P | N | N | N | P | N | N | X | X | N | X | X | N | 2 |
| Solomon et al. (2017) | Y | N | N | P | N | N | N | P | N | N | Y | N | N | Y | N | Y | 3 |
| Stephenson et al. (2018) | Y | N | N | Y | N | N | N | P | N | N | X | X | N | X | X | Y | 3.5 |
| Stewart et al. (2013) | Y | N | N | P | N | N | N | P | Y | N | N | Y | Y | N | N | N | 4 |
| Stover et al. (2009) | Y | N | N | P | N | N | N | P | N | N | X | X | N | X | X | N | 2 |
| Taylor and Harvey (2010) | Y | N | Y | P | N | Y | Y | N | N | N | Y | N | N | Y | Y | N | 3.5 |
| Tehrani (2016) | Y | N | N | P | N | N | N | N | N | N | Y | N | N | Y | N | N | 1.5 |
| Templer et al. (2017) | Y | N | N | P | N | Y | N | N | N | N | X | X | N | X | X | N | 2.5 |
| Thanhäuser et al. (2017) | Y | N | Y | Y | N | Y | N | N | Y | N | Y | N | N | Y | Y | Y | 5 |
| Trask et al. (2011) | Y | N | N | P | N | Y | N | N | N | N | Y | N | N | Y | Y | N | 2.5 |
| Troy et al. (2018) | Y | N | Y | P | Y | Y | N | N | Y | N | X | X | N | X | X | Y | 5.5 |
| Turner et al. (2007) | Y | Y | N | Y | Y | N | Y | Y | Y | N | Y | N | N | Y | N | Y | 8 |
| Uretsky and Hoffman (2017) | Y | N | N | P | N | Y | Y | N | N | N | Y | N | Y | N | N | N | 4.5 |
| Van Andel et al. (2014) | Y | N | N | P | N | N | N | N | N | N | Y | N | N | Y | X | N | 1.5 |
| Weiner et al. (2011) | Y | N | N | P | N | N | N | N | N | N | Y | N | N | N | Y | N | 1.5 |
| Wethington et al. (2008) | Y | N | Y | Y | N | Y | N | Y | P | N | Y | Y | N | Y | N | Y | 5.5 |
| Wilen (2014) | Y | Y | Y | Y | Y | Y | Y | Y | Y | Y | Y | Y | Y | Y | Y | N | 10 |
| Woodgate (2017) | Y | N | N | P | Y | Y | N | P | N | N | X | X | N | X | X | Y | 5 |
| Xiang (2013) | Y | N | Y | P | N | N | N | P | Y | N | X | X | Y | X | X | Y | 5 |
| Yelick (2017) | Y | N | N | P | N | N | N | P | N | N | X | X | N | X | X | N | 2 |
| Ziviani et al. (2012) | Y | P | N | P | Y | N | N | Y | Y | N | X | X | N | X | X | Y | 6 |

Key: Y=Yes, P=Partial Yes, N=No, X=Not applicable

**Appendix D. Quality assessment tools used in included reviews**

| **Review reference** | **QA tool name** | **Summary results** |
| --- | --- | --- |
| Altena et al. (2010) | US Preventive Services Task Force^1^ | Good n=0 Fair n=7 Poor n=7 |
| Bassuk et al. (2014) | EPHPP checklist | Strong n=0 Moderate n=2 Weak n=4 |
| Bee et al. (2014) | Cochrane Risk of Bias Tool | Low n=1 Unclear n=22 High n=14 |
| Beresford et al. (2008) | Jadad checklist; EPOC (Cochrane Effective Practice and Organisation of Care Group) checklist; Downs & Black^2^ | Jadad: 3/3 n=5 2/3 n=3 1/3 n=4 EPOC: 6/6 n=0 5/6 n=0 4/6 n=3 3/6 n=4 2/6 n=1 1.5/6 n=1 1/6 n=2 0/6 n=1 Downs & Black [incompletely reported]: >50% n=2 40-49% n=3 |
| British Columbia Centre (2013) | NICE Public Health Methods Manual (2^nd^ ed.) | High n=2 Medium n=9 (plus n=1 review) Low n=1 |
| Broning et al. (2012) | Scottish Intercollegiate Guidelines Network | Very good n=3 Good n=5 Modest n=2 Low n=3 |
| Coren et al. (2016) | Cochrane Risk of Bias Tool | Summary measure NR |
| Evans et al. (2017) | Cochrane Risk of Bias Tool | Summary measure NR |
| Everson-Hock et al. (2011) | NICE Public Health Methods Manual (1^st^ ed.) | High n=1 Medium n=3 Low n=3 |
| Everson-Hock et al. (2012) | NICE Public Health Methods Manual (1^st^ ed.) | High n=0 Medium n=3 Low n=3 |
| Fraser et al. (2013) | Review-specific tool | Summary measure NR |
| Howarth et al. (2016) | Cochrane Risk of Bias Tool | Low n=2 High n=4 Unclear n=7 |
| Jones et al. (2008) | NICE Public Health Methods Manual (1^st^ ed.) | High n=0 Medium n=3 Low n=2 |
| Kemmis-Riggs et al. (2018) | Cochrane Risk of Bias Tool | Low risk on N indices: 6/6 n=1 5/6 n=0 4/6 n=3 3/6 n=3 2/6 n=6 0/6 or 1/6 n=4 |
| Kinsey and Schlosser (2013) | Downs & Black^2^ | ≥70% n=19/22 |
| Leenarts et al. (2013) | Cochrane Risk of Bias Tool | Summary measure NR |
| Loechner et al. (2017) | Scottish Intercollegiate Guidelines Network | 8-10/10 n=11 6-7/10 n=3 |
| Macdonald et al. (2012) | Cochrane Risk of Bias Tool | Summary measure NR |
| Macdonald et al. (2016) | Cochrane Risk of Bias Tool | Summary measure NR |
| Montgomery et al. (2009) | [No QA reported] | – |
| Naranbhai et al. (2011) | Cochrane Risk of Bias Tool | Summary measure NR |
| Parker and Turner (2013) | Cochrane Risk of Bias Tool | Summary measure NR |
| Troy et al. (2018) | Review-specific tool (quality) | High n=6 Moderate n=11 Low n=12 |
| Turner et al. (2007) | Cochrane Risk of Bias Tool | Summary measure NR |
| Wethington et al. (2008) | Guide to Community Preventive Services^3^ | Good n=13 Fair n=17 |
| Wilen (2014) | Cochrane Risk of Bias Tool | Summary measure NR |
| Ziviani et al. (2012) | Physiotherapy Evidence Database (PEDro) Scale^4^; Downs & Black^2^ | PEDro: 5/11 n=2 Downs & Black: 17/28 n=1 14/28 n=1 |

1. Harris RP, Helfand M, Woolf SH, et al. Current methods of the US Preventive Services Task Force: a review of the process. American Journal of Preventive Medicine 2001;20(3):21-35.

2. Downs SH, Black N. The feasibility of creating a checklist for the assessment of the methodological quality both of randomised and non-randomised studies of health care interventions. Journal of Epidemiology & Community Health 1998;52(6):377-84.

3. Briss PA, Zaza S, Pappaioanou M, et al. Developing an evidence-based Guide to Community Preventive Services—methods. American Journal of Preventive Medicine 2000;18(1):35-43.

4. Maher CG, Sherrington C, Herbert RD, Moseley AM, Elkins M. Reliability of the PEDro scale for rating quality of randomized controlled trials. Physical Therapy 2003;83(8):713-21.

**References**

Al CMW, Stams GJJM, Bek MS, et al. A meta-analysis of intensive family preservation programs: Placement prevention and improvement of family functioning. Children and Youth Services Review 2012;34(8):1472-79.

Altena AM, Brilleslijper-Kater SN, Wolf JL. Effective interventions for homeless youth: a systematic review. American Journal of Preventive Medicine 2010;38(6):637-45.

Bassuk EL, DeCandia CJ, Tsertsvadze A, et al. The effectiveness of housing interventions and housing and service interventions on ending family homelessness: A systematic review. American Journal of Orthopsychiatry 2014;84(5):457-74.

Bee P, Bower P, Byford S, et al. The clinical effectiveness, cost-effectiveness and acceptability of community-based interventions aimed at improving or maintaining quality of life in children of parents with serious mental illness: a systematic review. Health Technology Assessment 2014;18(8).

Beresford B, Clarke S, Gridley K, et al. Technical report for SCIE research review on access, acceptability and outcomes of services/interventions to support parents with mental health problems and their families. York: Social Policy Research Unit, 2008.

Bergman A-S, Axberg U, Hanson E. When a parent dies: A systematic review of the effects of support programs for parentally bereaved children and their caregivers. BMC Palliative Care 2017;17:1-15.

British Columbia Centre of Excellence for Women’s Health. Review of interventions to identify, prevent, reduce and respond to domestic violence. London: National Institute for Health and Care Excellence, 2013.

Broning S, Kumpfer K, Kruse K, et al. Selective prevention programs for children from substance-affected families: a comprehensive systematic review. Substance Abuse Treatment, Prevention, & Policy 2012;7:23.

Byrne N. Systematic review of speech and language therapy outcomes for children who are in Out of Home Care (OOHC). Speech Language and Hearing 2017;20(1):57-61.

Calhoun S, Conner E, Miller M, et al. Improving the outcomes of children affected by parental substance abuse: a review of randomized controlled trials. Substance Abuse & Rehabilitation 2015;6:15-24.

Chen CY-C, Panebianco A. Interventions for young bereaved children: A systematic review and implications for school mental health providers. Child & Youth Care Forum 2018;47(2):151-71.

Chronis-Tuscano A, Wang CH, Woods KE, et al. Parent ADHD and evidence-based treatment for their children: Review and directions for future research. Journal of Abnormal Child Psychology 2017;45(3):501-17.

Corcoran J, Pillai V. A meta-analysis of parent-involved treatment for child sexual abuse. Research on Social Work Practice 2008;18(5):453-64.

Coren E, Hossain R, Pardo P, et al. Interventions for promoting reintegration and reducing harmful behaviour and lifestyles in street-connected children and young people. Cochrane Database of Systematic Reviews 2016(1):CD009823.

Cunha LM. The efficacy of therapeutic interventions for adolescent maltreatment victims: A meta-analysis. DPsych thesis, Alliant International University, 2008.

Currier JM, Holland JM, Neimeyer RA. The effectiveness of bereavement interventions with children: A meta-analytic review of controlled outcome research. Journal of Clinical Child and Adolescent Psychology 2007;36(2):253-59.

Davies BR, Allen NB. Trauma and homelessness in youth: Psychopathology and intervention. Clinical Psychology Review 2017;54:17-28.

Dawson A, Jackson D. The primary health care service experiences and needs of homeless youth: A narrative synthesis of current evidence. Contemporary Nurse 2013;44(1):62-75.

Dorrepaal E, Thomaes K, Hoogendoorn AW, et al. Evidence-based treatment for adult women with child abuse-related Complex PTSD: A quantitative review. European Journal of Psychotraumatology 2014;5:23613.

Downes MJ, Lakhani A, Maujean A, et al. Evidence for using farm care practices to improve attachment outcomes in foster children: A systematic review. British Journal of Social Work 2016;46(5):1241-48.

Ehring T, Welboren R, Morina N, et al. Meta-analysis of psychological treatments for posttraumatic stress disorder in adult survivors of childhood abuse. Clinical Psychology Review 2014;34(8):645-57.

Evans R, Brown R, Rees G, et al. Systematic review of educational interventions for looked-after children and young people: Recommendations for intervention development and evaluation. British Educational Research Journal 2017;43(1):68-94.

Everson-Hock ES, Jones R, Guillaume L, et al. Supporting the transition of looked-after young people to independent living: a systematic review of interventions and adult outcomes. Child: Care, Health & Development 2011;37(6):767-79.

Everson-Hock ES, Jones R, Guillaume L, et al. The effectiveness of training and support for carers and other professionals on the physical and emotional health and well-being of looked-after children and young people: a systematic review. Child: Care, Health & Development 2012;38(2):162-74.

Forsman H, Vinnerljung B. Interventions aiming to improve school achievements of children in out-of-home care: A scoping review. Children and Youth Services Review 2012;34(6):1084-91.

Fraser JG, Lloyd S, Murphy R, et al. A comparative effectiveness review of parenting and trauma-focused interventions for children exposed to maltreatment. Journal of Developmental & Behavioral Pediatrics 2013;34(5):353-68.

Furr-Roeske CM. Parenting interventions for children exposed to intimate partner violence. DNP thesis, University of South Carolina, 2011.

Gunlicks ML, Weissman MM. Change in child psychopathology with improvement in parental depression: A systematic review. Journal of the American Academy of Child & Adolescent Psychiatry 2008;47(4):379-89.

Hackett SM. Effectiveness of domestic violence victims interventions: A meta-analysis. PhD thesis, University of Oklahoma, 2013.

Hambrick EP, Oppenheim-Weller S, N'Zi A, et al. Mental health interventions for children in foster care: A systematic review. Children & Youth Services Review 2016;70:65-77.

Harvey ST, Taylor JE. A meta-analysis of the effects of psychotherapy with sexually abused children and adolescents. Clinical Psychology Review 2010;30(5):517-35.

Herbert JL, Bromfield L. Evidence for the efficacy of the Child Advocacy Center model. Trauma, Violence & Abuse 2016;17(3):341-57.

Hetzel-Riggin MD, Brausch AM, Montgomery BS. A meta-analytic investigation of therapy modality outcomes for sexually abused children and adolescents: An exploratory study. Child Abuse & Neglect 2007;31(2):125-41.

Holtzhausen L, Ross A, Perry R. Working on trauma: A systematic review of TF-CBT work with child survivors of sexual abuse. Social Work / Maatskaplike Werk 2016;52(4):511-24.

Hooker L, Kaspiew R, Taft A. Domestic and family violence and parenting: mixed methods insights into impact and support needs. Sydney: Australia’s National Research Organisation for Women’s Safety, 2016.

Howarth E, Moore THM, Welton NJ, et al. IMPRoving Outcomes for children exposed to domestic ViolencE (IMPROVE): an evidence synthesis. Public Health Resarch 2016;4(10).

Jensen de López K, Knudsen HS, Hansen TGB. What is measured in bereavement treatment for children and adolescents? A systematic literature review. Illness, Crisis & Loss 2017;0.

Jones R, Everson-Hock ES, Guillaume L, et al. The effectiveness of interventions aimed at improving access to health and mental health services for looked after children and young people. Sheffield: School of Health and Related Research, 2008.

Journot-Reverbel K, Raynaud JP, Bui E, et al. Support groups for children and adolescents bereaved by suicide: Lots of interventions, little evidence. Psychiatry Research 2017;250:253-55.

Kanine RM, Tunno AM, Jackson Y, et al. Therapeutic day treatment for young maltreated children: A systematic literature review. Journal of Child and Adolescent Trauma 2015;8(3):187-99.

Kemmis-Riggs J, Dickes A, McAloon J. Program components of psychosocial interventions in foster and kinship care: A systematic review. Clinical Child & Family Psychology Review 2018;21(1):13-40.

Kerr L, Cossar J. Attachment interventions with foster and adoptive parents: A systematic review. Child Abuse Review 2014;23(6):426-39.

Kim S, Noh D, Kim H. A summary of selective experimental research on psychosocial interventions for sexually abused children. Journal of Child Sexual Abuse 2016;25(5):597-617.

Kinsey D, Schlosser A. Interventions in foster and kinship care: a systematic review. Clinical Child Psychology & Psychiatry 2013;18(3):429-63.

Korotana LM, Dobson KS, Pusch D, et al. A review of primary care interventions to improve health outcomes in adult survivors of adverse childhood experiences. Clinical Psychology Review 2016;46:59-90.

Kowalik J, Weller J, Venter J, et al. Cognitive behavioral therapy for the treatment of pediatric posttraumatic stress disorder: A review and meta-analysis. Journal of Behavior Therapy and Experimental Psychiatry 2011;42(3):405-13.

Leenarts LE, Diehle J, Doreleijers TA, et al. Evidence-based treatments for children with trauma-related psychopathology as a result of childhood maltreatment: a systematic review. European Child & Adolescent Psychiatry 2013;22(5):269-83.

Leve LD, Harold GT, Chamberlain P, et al. Practitioner review: Children in foster care - vulnerabilities and evidence-based interventions that promote resilience processes. Journal of Child Psychology & Psychiatry & Allied Disciplines 2012;53(12):1197-211.

Liabo K, Gray K, Mulcahy D. A systematic review of interventions to support looked-after children in school. Child & Family Social Work 2013;18(3):341-53.

Loechner J, Starman K, Galuschka K, et al. Preventing depression in the offspring of parents with depression: A systematic review and meta-analysis of randomized controlled trials. Clinical Psychology Review 2017;29:29.

Macdonald G, Higgins JPT, Ramchandani P, et al. Cognitive-behavioural interventions for children who have been sexually abused: a systematic review. Cochrane Database of Systematic Reviews 2012;5:CD001930.

Macdonald G, Livingstone N, Hanratty J, et al. The effectiveness, acceptability and cost-effectiveness of psychosocial interventions for maltreated children and adolescents: an evidence synthesis. Health Technology Assessment 2016;20(69):1-508.

Mannay D, Staples E, Hallett S, et al. Understanding the educational experiences and opinions, attainment, achievement and aspirations of looked after children in Wales. Cardiff: Department for Education and Skills, Welsh Government, 2015.

Marsh DD. A systematic review and meta-analysis of randomized clinical trials of evidence-based practices through measured change of behavior for children in foster care. DPsych thesis, Chicago School of Professional Psychology, 2017.

McDonnell CJ, Garbers SV. Adverse Childhood Experiences and obesity: Systematic review of behavioral interventions for women. Psychological Trauma: Theory, Pesearch, Practice and Policy 2017;10(4):387-95.

McMillan AS, Barlow J, Stewart-Brown SL, et al. Systematic review of interventions for the secondary prevention and treatment of emotional abuse of children by primary carers. Warwick: Warwick Medical School, 2008.

Miffitt LA. State of the science: Group therapy interventions for sexually abused children. Archives of Psychiatric Nursing 2014;28(3):174-9.

Montgomery P, Gardner F, Ramchandani P, et al. Systematic reviews of interventions following physical abuse: helping practitioners and expert witnesses improve the outcomes of child abuse. Oxford: Centre for Evidence-Based Intervention, 2009.

Naranbhai V, Abdool K, Meyer-Weitz A. Interventions to modify sexual risk behaviours for preventing HIV in homeless youth. Cochrane Database of Systematic Reviews 2011(1):CD007501.

Niccols A, Milligan K, Smith A, et al. Integrated programs for mothers with substance abuse issues and their children: A systematic review of studies reporting on child outcomes. Child Abuse & Neglect 2012;36(4):308-22.

O'Haire ME, Guerin NA, Kirkham AC. Animal-Assisted Intervention for trauma: A systematic literature review. Frontiers in Psychology 2015;6:1121.

Parker B, Turner W. Psychoanalytic/psychodynamic psychotherapy for children and adolescents who have been sexually abused. Cochrane Database of Systematic Reviews 2013(7):CD008162.

Passarela CDM, Mendes DD, De Jesus M. A systematic review to study the efficacy of cognitive behavioral therapy for sexually abused children and adolescents with posttraumatic stress disorder. Revista de Psiquiatria Clinica 2010;37(2):63-73.

Phillips SD, Gleeson JP, Waites-Garrett M. Substance-abusing parents in the criminal justice system: Does substance abuse treatment improve their children's outcomes? Journal of Offender Rehabilitation 2009;48(2):120-38.

Poli CF, Molgora S, Marzotto C, et al. Group interventions for children having separated parents: A systematic narrative review. Journal of Divorce & Remarriage 2017;58(8):559-83.

Premji S, Benzies K, Serrett K, et al. Research-based interventions for children and youth with a Fetal Alcohol Spectrum Disorder: Revealing the gap. Child: Care, Health & Development 2007;33(4):389-97.

Reupert AE, Cuff R, Drost L, et al. Intervention programs for children whose parents have a mental illness: A review. Medical Journal of Australia 2013;199(3 Suppl):S18-22.

Rizo CF, Macy RJ, Ermentrout DM, et al. A review of family interventions for intimate partner violence with a child focus or child component. Aggression and Violent Behavior 2011;16(2):144-66.

Roberts L, Maxwell N, Rees P, et al. Improving well-being and outcomes for looked after children in Wales: A context sensitive review of interventions. Adoption and Fostering 2016;40(4):309-24.

Rosner R, Kruse J, Hagl M. A meta-analysis of interventions for bereaved children and adolescents. Death Studies 2010;34(2):99-136.

Rubin A, Washburn M, Schieszler C. Within-group effect-size benchmarks for trauma-focused Cognitive Behavioral Therapy with children and adolescents. Research on Social Work Practice 2017;27(7):789-801.

Ruff S, McComb JL, Coker CJ, et al. Behavioral couples therapy for the treatment of substance abuse: A substantive and methodological review of O'Farrell, Fals-Stewart, and colleagues' program of research. Family Process 2010;49(4):439-56.

Sanchez-Meca J, Rosa-Alcazar AI, Lopez-Soler C. The psychological treatment of sexual abuse in children and adolescents: A meta-analysis. International Journal of Clinical and Health Psychology 2011;11(1):67-93.

Siegenthaler E, Munder T, Egger M. Effect of preventive interventions in mentally ill parents on the mental health of the offspring: Systematic review and meta-analysis. Journal of the American Academy of Child & Adolescent Psychiatry 2012;51(1):8-17.e8.

Silverman WK, Ortiz CD, Viswesvaran C, et al. Evidence-based psychosocial treatments for children and adolescents exposed to traumatic events. Journal of Clinical Child & Adolescent Psychology 2008;37(1):156-83.

Slesnick N, Dashora P, Letcher A, et al. A review of services and interventions for runaway and homeless youth: Moving forward. Children & Youth Services Review 2009;31(7):732-42.

Solomon DT, Niec LN, Schoonover CE. The impact of foster parent training on parenting skills and child disruptive behavior. Child Maltreatment 2017;22(1):3-13.

Stephenson LA, Beck K, Busuulwa P, et al. Perinatal interventions for mothers and fathers who are survivors of childhood sexual abuse. Child Abuse & Neglect 2018;80:9-31.

Stewart S, Leschied A, Dunnen W, et al. Treating mental health disorders for children in child welfare care: Evaluating the outcome literature. Child & Youth Care Forum 2013;42(2):131-54.

Stover CS, Meadows AL, Kaufman J. Interventions for intimate partner violence: Review and implications for evidence-based practice. Professional Psychology - Research and Practice 2009;40(3):223-33.

Taylor JE, Harvey ST. A meta-analysis of the effects of psychotherapy with adults sexually abused in childhood. Clinical Psychology Review 2010;30(6):749-67.

Tehrani E. Comparisons between traditional and contemporary treatment modalities for sexually abused children and adolescents: A meta-analysis. PhD thesis, Chicago School of Professional Psychology, 2014.

Templer K, Matthewson M, Haines J, et al. Recommendations for best practice in response to parental alienation: Findings from a systematic review. Journal of Family Therapy 2017;39(1):103-22.

Thanhäuser M, Lemmer G, de Girolamo G, et al. Do preventive interventions for children of mentally ill parents work? Results of a systematic review and meta-analysis. Current Opinion in Psychiatry 2017;30(4):283-99.

Trask EV, Walsh K, Dilillo D. Treatment effects for common outcomes of child sexual abuse: A current meta-analysis. Aggression & Violent Behavior 2011;16(1):6-19.

Troy V, McPherson Kerri E, Emslie C, et al. The Feasibility, Appropriateness, Meaningfulness, and Effectiveness of Parenting and Family Support Programs Delivered in the Criminal Justice System: A Systematic Review. Journal of Child and Family Studies 2018:1-16.

Turner W, Macdonald G, Dennis JA. Behavioural and cognitive behavioural training interventions for assisting foster carers in the management of difficult behaviour. Cochrane Database of Systematic Reviews 2007;1:CD003760.

Uretsky MC, Hoffman JA. Evidence for group-based foster parent training programs in reducing externalizing child behaviors: A systematic review and meta-analysis. Journal of Public Child Welfare 2017;11(4-5):464-86.

Van Andel HWH, Grietens H, Strijker J, et al. Searching for effective interventions for foster children under stress: A meta-analysis. Child & Family Social Work 2014;19(2):149-55.

Weiner HA. Examining the effectiveness of psychological treatments and interventions for child maltreatment: A meta-analysis. DPsych thesis, Pace University, 2011.

Wethington HR, Hahn RA, Fuqua-Whitley DS, et al. The effectiveness of interventions to reduce psychological harm from traumatic events among children and adolescents: A systematic review. American Journal of Preventive Medicine 2008;35(3):287-313.

Wilen JS. A systematic review and network meta-analysis of psychosocial interventions for adults who are sexually abused as children. PhD thesis, Bryn Mawr College, 2014.

Woodgate RL, Morakinyo O, Martin KM. Interventions for youth aging out of care: A scoping review. Children and Youth Services Review 2017;82:280-300.

Xiang X. A review of interventions for substance use among homeless youth. Research on Social Work Practice 2013;23(1):34-45.

Yelick A. Research review: Independent living programmes: the influence on youth ageing out of care (YAO). Child & Family Social Work 2017;22(1):515-26.

Ziviani J, Feeney R, Cuskelly M, et al. Effectiveness of support services for children and young people with challenging behaviours related to or secondary to disability, who are in out-of-home care: A systematic review. Children and Youth Services Review 2012;34(4):758-70.
